# The Relation of Preoperative HbA1c Level With Intraoperative and Postoperative Complications in Type-2 Diabetic Patients: An Observational Study

**DOI:** 10.7759/cureus.64487

**Published:** 2024-07-13

**Authors:** Dhananj Shivganesh B. R., Habib Md R Karim, Nandkishore Agrawal, Mayank Kumar

**Affiliations:** 1 Anesthesiology and Critical Care, Post Graduate Institute of Medical Education and Research, Chandigarh, IND; 2 Anesthesiology, Critical Care, and Pain Medicine, All India Institute of Medical Sciences, Guwahati, IND; 3 Anesthesiology, Critical Care, and Pain Medicine, All India Institute of Medical Sciences, Raipur, IND

**Keywords:** elective general surgery, hyperglycemia, glycemic control, perioperative complications, hba1c, diabetes

## Abstract

Background

Perioperative dysglycemia increases morbidity and mortality, particularly among those with diabetes mellitus (DM), and elevated HbA1c levels, reflecting long-term blood glucose, are linked to poor healing and higher infection rates. This study investigates the link between preoperative HbA1c levels and perioperative outcomes in type-2 DM patients.

Methodology

This prospective observational study was conducted in India between January 2021 and April 2022. Sixty patients aged 18-60 with type-2 DM who underwent elective surgery under general anesthesia (GA) were included; the American Society of Anesthesiologists class >III and patients with severe organ failures were excluded. Participants were divided into two groups: A (HbA1c ≤7.5%) and B (HbA1c >7.5%). Data on preoperative vitals, intraoperative hemodynamics, and postoperative complications were collected. SPSS v23 was used for data analysis; p-value <0.05 was considered significant.

Results

The mean age of the participants was 48.22 years; males comprised 58.3%. Group A had a higher proportion of oral hypoglycemic agents. Group B showed higher maximum mean blood pressure and intraoperative blood sugar levels at one hour. Postoperatively, Group B had higher glucose levels, more prevalent hyperglycemia, and higher preoperative and postoperative blood urea levels. No significant differences were found in postoperative outcomes like acute kidney injury (AKI), leukocytopenia, leucocytosis, fever, and intensive care admission. Surgical site infection (SSI) incidence was higher in group B, though not statistically significant. Group B had more extended hospital stays.

Conclusion

Preoperative HbA1c above 7.5% was associated with impaired perioperative glycemic control and higher dysglycemic episodes. Higher preoperative HbA1c was found to be linked to increased postoperative hyperglycemia, AKI, intensive care admissions, and more extended hospital stays, though not statistically significant. SSI incidence was higher, highlighting its importance over preoperative HbA1c.

## Introduction

Perioperative blood glucose abnormalities, including hyperglycemia, hypoglycemia, and fluctuations, can increase mortality and complications in surgical patients [1,2]. Diabetes mellitus (DM) is a constellation of metabolic disorders primarily characterized by elevated blood glucose levels, i.e., hyperglycemia. These patients are particularly at risk for glycemic variations. Hyperglycemia during cardiac surgery, for instance, can escalate mortality and lead to renal and lung injuries, as well as atrial fibrillation [3]. Managing blood glucose levels is crucial during surgical procedures for diabetic patients to mitigate risks like perioperative ketoacidosis or hyperosmolar syndrome, impaired wound healing, and compromised leukocyte function [4,5]. Elevated HbA1c levels, reflecting average blood glucose over weeks, are associated with hindered healing and increased infection susceptibility. Poor preoperative glucose control, indicated by HbA1c levels, is an independent predictor of worse perioperative outcomes [6]. However, achieving optimal HbA1c levels pre-surgery can be challenging and time-consuming. Limited data exists on the correlation between preoperative HbA1c levels and intraoperative/postoperative complications, especially for non-cardiovascular and non-orthopedic surgeries.

Therefore, our study aimed to investigate the correlation of preoperative HbA1c levels with intraoperative and postoperative complications in type-2 DM patients. By understanding this relationship, we aimed to underscore the importance of measuring preoperative HbA1c levels in guiding surgical management for diabetic patients.

## Materials and methods

Study design and settings

The prospective observational study, which focused on adult patients, was conducted from January 2021 to April 2022 at an academic institute in India. The protocol was reviewed by the postgraduate thesis review committee, followed by the institute ethics committee, and approved for human studies. Informed consent was obtained from the participants, and recruitment was done after the study was registered in India's Clinical Trial Registry.

Participants

Patients planned for elective surgery were screened to fulfill inclusion criteria: patients aged between 18 and 60, of either male or female gender, with type-2 DM whose preoperative HbA1c levels were available within seven days, and normal fasting blood sugar levels during this period undergoing surgery under general anesthesia (GA) with laryngoscopy and endotracheal intubation (LETI). Exclusion criteria include patients unwilling to consent and those with hypertension, heart failure, arrhythmias, pregnancy, immunodeficiency, renal failure, thyroid disorders, or American Society of Anesthesiologists physical status (ASA-PS) class III and more.

Sample size

The project being a time-bound postgraduate thesis, we considered a convenient sampling of 60 participants. Furthermore, we considered HbA1c ≤7.5% as a cutoff to divide participants into groups: groups A (HbA1c ≤7.5%) and B (HbA1c >7.5%), and planned to recruit equal participants for each group, making the sample size 30 participants per group.

Anesthesia technique

The study was observational, and the investigators did not interfere with the anesthesia and analgesia techniques. However, in our institute, the practice prevalent for such elective surgical cases is balanced GA where Fentanyl or Morphine is used as an analgesic, Propofol is used as an intravenous anesthetic for induction, and Vecuronium or Rocuronium is used as a muscle relaxant. Maintenance is usually done with Isoflurane or Sevoflurane with a target age-adjusted minimum alveolar concentration of 1.1±0.1. Further analgesia supplements, both intraoperative and postoperative plans, usually follow multimodal analgesia with a combination of regional technique, paracetamol, and rescue analgesics. Reversal of neuromuscular blockade is done using Neostigmine and Glycopyrrolate. 

Data and outcome variable

Data were collected for preoperative baseline vitals, and intraoperative monitoring included assessing hemodynamic instability and hypoglycemic episodes. Postoperatively, parameters such as blood glucose, ketone bodies, electrocardiogram, leukocyte count, renal function, and wound status were monitored for complications. The surgical site infection (SSI) wound was graded per the Southhampton grading system [7].

Data management and statistical analysis

Data were entered into Microsoft Excel and analyzed using Statistical Package for the Social Sciences v23 (IBM Corporation, NY, USA). Descriptive statistics were utilized to present means/standard deviations and medians/ interquartile ranges (IQR) for continuous variables and frequencies/percentages for categorical variables. A density plot is used to show data distribution. Group comparisons utilized independent sample t-tests for continuous data and chi-squared tests for categorical data. Non-normally distributed data were analyzed using the Wilcoxon test. Correlation between continuous variables was explored using Pearson's correlation for normally distributed data and Spearman's correlation for non-normally distributed data. Statistical significance was set at <0.05. Paired analysis for continuous variables was conducted using the Paired t-test or Wilcoxon signed rank test, while the Friedman test was employed to compare multiple continuous variables.

## Results

The entire 60 participants enrolled completed the study. The mean age of participants was 48.22 years, with a slightly higher representation of males 35 (58.3%). Most participants belonged to the American Society of Anesthesiologists Physical Status (ASA-PS) class-2 58(93.3%). The mean body mass index (BMI) was 23.84 kg/m², with 46 (76.7%) participants on oral hypoglycemic agents; the data are shown in Table 1.

**Table 1:** Basic clinico-demographic data. SD: standard deviation, IQR: interquartile range, ASA-PS: American Society of Anesthesiologists physical status, N: total number

Parameters (N=60)	Value
Age (Years) Mean ± SD	48.22±7.42
Male n (%)	35 (58.3%)
Female n (%)	25 (41.7%)
BMI (Kg/m²) Mean ± SD	23.84±3.09
ASA PS-II	56 (93.3%)
ASA PS-III	4 (6.7%)
Duration of DM (Years) Median (IQR)	5.0 (3.0-6.0)
Fasting Sugar Mean ± SD	108.03±15.51

Significant differences were observed between the groups concerning the usage of oral hypoglycemic agents, with a higher proportion in Group A (96.7%) compared to Group B (56.7%). The study found no significant difference between the groups regarding demographic variables; however, there was a significant difference in preoperative HbA1c levels between the groups, with Group B exhibiting higher median HbA1c levels. Furthermore, no significant difference was observed between the groups concerning type-2 DM or surgery duration. The baseline characteristics are presented in Table 2.

**Table 2:** Comparison of baseline characteristics of the participants among the group presented as either mean ± SD or number and percentage scale and analyzed using T-test (T) or Chi-square (χ2) or Wilcoxon test (W); p-value <0.05 was considered significant. ^#^Chi-square test ^@^Wilcoxon test ASA-PS: American Society of Anesthesiologists physical status, BMI: body mass index, OHA: oral hypoglycemic agent, SD: standard deviation, N: total number

Parameters	Group A (HbA1c ≤7.5 gm%) (N=30)	Group B (HbA1c >7.5 gm%) (N=30)	T/χ2/W	p-value
Age (years)	48.23±7.09	48.20±7.85	0.017	0.986
Male n (%)	15 (50.0%)	20 (66.7%)	1.714	0.190^#^
Female n (%)	15 (50.0%)	10 (33.3%)		
BMI (kg/m^2^)	23.21±2.67	24.47±3.39	-1.600	0.115
ASA-PS II	29 (96.7%)	27 (90.0%)	1.071	0.612^#^
ASA-PS III	1 (3.3%)	3 (10.0%)		
Duration of DM (years)	4.90±3.12	5.50±4.27	420.000	0.660
Taking OHA	29 (96.7%)	17 (56.7%)	13.416	<0.001
Insulin (total units/day)	26.80±7.29	30.45±13.59	-0.819	0.429^@^
Preop HbA1c	7.14±0.24	9.14±1.32	0.000	<0.001
Preop Bl urea	18.97±6.95	27.13±14.00	278.000	0.011
Preop S. creatinine	0.86±0.17	0.96±0.26	-1.692	0.097^@^
Duration of surgery (hours)	3.67±1.84	4.03±1.53	359.500	0.179

The mean maximum pulse rate in Group A was 98.17±15.53, while in Group B, it was slightly higher at 101.83±14.60. Similarly, the mean minimum pulse rate in Group A was 73.20±14.05; in Group B, it was 74.80±12.61. No significant differences were found between the groups regarding maximum and minimum pulse rates. However, there was a significant difference in maximum mean arterial pressure (MAP) between the groups, with Group B having a higher mean of 101.50±9.19 compared to Group A's 96.80±8.88 mmHg; p-value: 0.04.

Intraoperative blood sugar levels at two, three, four, and five hours were comparable among the groups. However, group B's mean ± SD blood sugar levels were significantly higher at one hour (Table 3).

**Table 3:** Intraoperative blood sugar levels among the group presented as mean ± SD and compared using the Wilcoxon test; p-value <0.05 was considered significant. SD: standard deviation, N: total number

Timeline	Group A (HbA1c ≤7.5 gm%) (N=30)	Group B (HbA1c >7.5 gm% ) (N=30)	W	p-value
1h Mean ± SD	140.47±29.09	161.93±46.66	298.500	0.026
2h Mean ± SD	141.13±31.44	158.12±37.28	189.500	0.067
3h Mean ± SD	146.90±26.59	154.87±34.01	74.000	0.978
4h Mean ± SD	147.00±44.17	158.12±48.23	-0.426	0.680
5h Mean ± SD	171.50±14.85	178.75±50.41	-0.266	0.804

**Figure 1:**
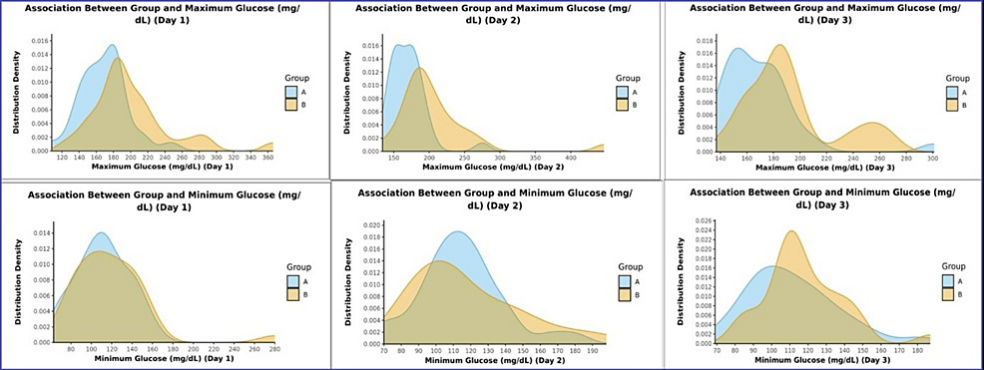
Density plot distribution of the maximum and minimum blood sugar levels (mg/dL) on postoperative days one to three.

Regarding blood sugar levels, Group B exhibited higher median glucose levels on day one postoperatively (190.5 mg/dL, IQR=180-213.5) compared to Group A (167.5 mg/dL, IQR=151-183), and this difference was statistically significant. Additionally, on day two postoperatively, Group B had significantly higher maximum glucose levels (206.70 mg/dL, IQR=178.75-218.25) compared to Group A (171.5 mg/dL, IQR=150.5-181.5). Postoperative hyperglycemia was more prevalent in Group B (26; 93.3%) than in Group A (16; 53.3%). Figure 1 presents the density plot distribution of the maximum and minimum glucose levels (mg/dL) on postoperative days one to three.

Group B exhibited higher preoperative blood urea levels (median=25 mg/dL, IQR=17.75-33.75) compared to Group A (median=18.5 mg/dL, IQR=14.25-24), and a similar pattern persisted in the postoperative period. Group B exhibited significantly higher mean levels of postoperative blood urea (29.13±13.91 mg/dL) than Group A (19.77±6.7 mg/dL, p=0.002). However, the preoperative serum creatinine levels did not significantly differ between the groups (Group A: 0.86±0.17; Group B: 0.96±0.26 mg/dL; p=0.097). Postoperatively, the two groups had no significant difference in serum creatinine levels (Group A: 0.89±0.18; Group B: 0.99±0.29 mg/dL; p=0.114).

Furthermore, the association between preoperative HbA1c levels and various postoperative outcomes, such as AKI, leukocytopenia, leucocytosis, fever within three days of surgery, ICU admission, and SSI wound grades, did not yield significant differences between the groups. Notably, while Group B had a higher incidence of AKI (6.7%) compared to Group A (0.0%), the differences were not statistically significant (p=0.492). Similarly, leukocytopenia, leucocytosis, fever, ICU admission, and SSI did not significantly differ between the two groups. Additionally, wound scoring revealed variations between the groups. Group B displayed a broader distribution of scores compared to Group A. SSI wound grade three and above features were more prevalent in Group B (4; 13.3%) than in Group A (0; 0.0%). However, these differences between the groups for Southhampton grading did not reach statistical significance (p=0.702, Table 4).

**Table 4:** Southampton wound score for SSI among the groups presented in number and percentage scale compared using Chi-square test; p-value <0.05 was considered significant. SSI: surgical site infection

SSI wound grade	Group A (HbA1c ≤7.5 gm%)	Group B (HbA1c >7.5 gm%)	Total	χ2	p-value
0	7 (58.3%)	6 (31.6%)	13 (41.9%)	6.354	0.702
1A	3 (25.0%)	2 (10.5%)	5 (16.1%)		
1C	1 (8.3%)	3 (15.8%)	4 (12.9%)		
2A	1 (8.3%)	2 (10.5%)	3 (9.7%)		
2B	0 (0.0%)	2 (10.5%)	2 (6.5%)		
3A	0 (0.0%)	1 (5.3%)	1 (3.2%)		
3B	0 (0.0%)	2 (10.5%)	2 (6.5%)		
4A	0 (0.0%)	1 (5.3%)	1 (3.2%)		
Total	12 (100.0%)	19 (100.0%)	31 (100.0%)		

The comparison of hospital stay duration between Group A and Group B revealed no significant difference in their distributions. Group B had a slightly longer mean duration of hospital stay (8.33±4.92 days) than Group A (6.63±3.43 days). The median duration of hospital stay was also slightly higher in Group B (eight days, IQR=4-10.75) than in Group A (six days, IQR=4-8). However, the Wilcoxon-Mann-Whitney U Test did not detect a statistically significant difference between the two groups; p=0.219. Despite the lack of statistical significance, a small effect size (Point-Biserial correlation=0.2) was observed, suggesting a subtle association between the groups' duration of hospital stay and their respective treatments or conditions. This finding implies that while there may be a trend toward more extended hospital stays in Group B, it is not significantly different from Group A based on the available data. A comparison of the two groups in terms of intra- and postoperative morbidities is presented in Table 5, and the odds for the events are presented in Table 6.

**Table 5:** Comparison of the two groups regarding intra- and postoperative morbidities presented as mean ± SD or number and percentage scale and tested using Chi-square (χ2) or Wilcoxon test (W); p-value <0.05 was considered significant. ^#^Wilcoxon test SD: standard deviation, N: total number, AKI: acute kidney injury, SSI: surgical site infection, MAP: mean arterial pressure

Parameters	Group A (HbA1c ≤7.5 gm%) (N=30)	Group B (HbA1c >7.5 gm%) (N=30)	χ2/W	p-value
Maximum heart rate	98.17±15.53	101.83±14.60	-0.942	0.350^#^
Minimum Heart rate	73.20±14.05	74.80±12.61	-0.464	0.644^#^
Maximum MAP	96.80±8.88	101.50±9.19	-2.014	0.049^#^
Minimum MAP	73.53±10.79	72.17±7.43	0.571	0.570^#^
Tachycardia	10 (33.3%)	14 (46.7%)	1.111	0.292
Bradycardia	4 (13.3%)	6 (20.0%)	0.480	0.488
Hypertension	2 (6.7%)	6 (20.0%)	2.31	0.254
Hypotension	17 (56.7%)	18 (60.0%)	0.069	0.793
Intraoperative hyperglycemia	4 (13.3%)	10 (33.3%)	2.45	0.117
Intraoperative hypoglycemia	4 (13.3%)	9 (30.0%)	2.45	0.117
Postoperative hypertension	2 (6.7%)	5 (16.7%)	1.456	0.424
Postoperative hypotension	3 (10.0%)	2 (6.7%)	0.218	1.000
Postoperative hyperglycemia	16 (53.3%)	28 (93.3%)	12.273	<0.001
Postoperative hypoglycemia	6 (20.0%)	5 (16.7%)	0.111	0.739
AKI	0 (0.0%)	2 (6.7%)	2.069	0.492
Blood urea (mg/dL) (Postoperative)	19.77±6.72	29.13±13.91	-3.32	0.002^#^
Serum creatinine (mg/dL) (Postoperative)	0.89±0.18	0.99±0.29	343.00	0.114^#^
Leukocytopenia	2 (6.7%)	0 (0.0%)	2.069	0.492
Leukocytosis	8 (26.7%)	15 (50.0%)	3.455	0.063
Fever within postoperative three days	5 (16.7%)	7 (23.3%)	0.417	0.519
Intensive care admission	7 (23.3%)	9 (30.0%)	0.341	0.559
SSI (overall)	7 (23.3%)	13 (43.3%)	2.700	0.100
Duration of hospital stay (days)	6.63±3.43	8.33±4.92	367.00	0.219^#^

**Table 6:** Odds of intra- and postoperative adverse events and outcomes. CI: confidence interval, OR: odds ratio, RR: relative risk, SSI: surgical site infection

Risk factor	Outcome	OR (95% CI)	RR (95% CI)
Preop HbA1c ≤7.5%	Intraoperative tachycardia	0.57 (0.2-1.62)	0.71 (0.37-1.33)
Preop HbA1c >7.5%		1.75 (0.62-4.97)	1.4 (0.75-2.67)
Preop HbA1c ≤7.5%	Intraoperative bradycardia	0.62 (0.15-2.45)	0.67 (0.22-1.99)
Preop HbA1c >7.5%		1.62 (0.41-6.47)	1.5 (0.5-4.58)
Preop HbA1c ≤7.5%	Intraoperative hypertension	0.29 (0.05-1.55)	0.33 (0.08-1.32)
Preop HbA1c >7.5%		3.5 (0.65-18.98)	3 (0.76-12.39)
Preop HbA1c ≤7.5%	Intraoperative hypotension	0.87 (0.31-2.43)	0.94 (0.61-1.46)
Preop HbA1c >7.5%		1.15 (0.41-3.2)	1.06 (0.68-1.65)
Preop HbA1c ≤7.5%	Intraoperative hyperglycemia	0.31 (0.08-1.13)	0.4 (0.14-1.06)
Preop HbA1c >7.5%		3.25 (0.89-11.9)	2.5 (0.94-6.96)
Preop HbA1c ≤7.5%	Intraoperative hypoglycemia	0.36 (0.1-1.33)	0.44 (0.16-1.2)
Preop HbA1c >7.5%		2.79 (0.75-10.33)	2.25 (0.83-6.37)
Preop HbA1c ≤7.5%	Postoperative hypertension	0.36 (0.06-2.01)	0.4 (0.09-1.64)
Preop HbA1c >7.5%		2.8 (0.5-15.73)	2.5 (0.61-10.65)
Preop HbA1c ≤7.5%	Postoperative hypotension	1.56 (0.24-10.05)	1.5 (0.32-7.16)
Preop HbA1c >7.5%		0.64 (0.1-4.15)	0.67 (0.14-3.14)
Preop HbA1c ≤7.5%	Postoperative hyperglycemia	0.08 (0.02-0.41)	0.57 (0.38-0.78)
Preop HbA1c >7.5%		12.25 (2.46-60.91)	1.75 (1.29-2.6)
Preop HbA1c ≤7.5%	Postoperative hypoglycemia	1.25 (0.34-4.64)	1.2 (0.43-3.38)
Preop HbA1c >7.5%		0.8 (0.22-2.97)	0.83 (0.3-2.33)
Preop HbA1c ≤7.5%	Leukocytosis	0.36 (0.12-1.07)	0.53 (0.26-1.03)
Preop HbA1c >7.5%		2.75 (0.93-8.1)	1.88 (0.97-3.8)
Preop HbA1c ≤7.5%	Postoperative fever	0.66 (0.18-2.36)	0.71 (0.26-1.91)
Preop HbA1c >7.5%		1.52 (0.42-5.47)	1.4 (0.52-3.81)
Preop HbA1c ≤7.5%	Intensive care admission	0.71 (0.22-2.25)	0.78 (0.34-1.77)
Preop HbA1c >7.5%		1.41 (0.45-4.45)	1.29 (0.56-2.97)
Preop HbA1c ≤7.5%	SSI	0.4 (0.13-1.21)	0.54 (0.25-1.12)
Preop HbA1c >7.5%		2.51 (0.83-7.64)	1.86 (0.89-4.03)

## Discussion

The present study's results indicated that patients who had higher HbA1c levels (≥7.5%) measured within seven days preoperatively also had higher blood sugar levels one hour before the commencement of surgery. However, intraoperative blood sugar levels were similar among both groups. This agrees with the 2014 study by Subramonium et al. [8].

Postoperatively, hyperglycemic episodes were significantly more prevalent in Group B, with the incidence higher on postoperative day one compared to days two and three, in coherence with the Perna et al. study [9]. The incidence of AKI was higher in Group B, though not statistically significant, contrary to the 2017 retrospective study by Kocogullari et al. [10].

Both groups did not statistically differ in postoperative rhythm changes, maximum and minimum pulse rates during the intraoperative and postoperative period, and mean arterial pressures. Two studies by Adams et al. and Strahan et al. suggested a cardioprotective effect conveyed by elevated HbA1c, while another study by Knapik et al. showed increased cardiovascular events in patients with elevated preoperative HbA1c levels [11-13].

The requirement of postoperative ICU admission was not significantly different in both groups. The incidence of SSI was higher in Group B patients with preoperative HbA1c levels of more than 7.5%, though not statistically significant. Our study found a significantly increased incidence of SSI in patients with preoperative HbA1c levels above 8.85%. The incidence of SSI was found in those patients who had poor postoperative glycemic control, irrespective of their preoperative HbA1c level. In the 2018 meta-analysis and systematic review by Shohat et al., an analysis of 10 studies suggested that elevated HbA1C levels were associated with a higher risk of SSI after total joint arthroplasty [14]. However, it was concluded that the pooled data did not support the conventional HbA1c 7% cutoff for risk stratification. Another 2014 study by Hikata et al. showed that patients with DM had a higher rate of SSI (six of 36 patients, 16.7%) than patients without DM (10 of 309 patients, 3.2%) [15]. Although the perioperative serum glucose level did not differ, the preoperative HbA1c value was significantly higher in the patients who developed SSI (7.6%) than in those who did not (6.9%). SSI developed in 0.0% of the patients with controlled diabetes (HbA1c <7.0%) and 35.3% of the patients with uncontrolled diabetes (HbA1c ≥7.0%). In our study, SSI wounds with pus and tissue necrosis corresponding to Southhampton grade three and above were only noted in patients with preoperative HbA1c > 7.5%.

We analyzed the duration of postoperative hospital stay in both groups of patients. The mean duration of hospital stay in Group A patients was 6.63 days, while for Group B patients was 8.33 days. No association was found between higher preoperative HbA1c levels and increased hospital stay. Most previous studies on this aspect were conducted on cardiac surgeries and are retrospective, suggesting that further prospective studies are needed in non-cardiac surgeries for comparative analysis [16,17].

The cutoff for defining higher levels of HbA1c needs to be more consistently defined, and taking a higher value than the one we have taken in the present study might impact the outcome. The current management has remained widely variable [18].

Although the present study has the strengths of a good amount of data and robust analysis, its limitations also need to be considered. It was an observational study with a limited sample size and conducted in a single center. Further multicentric prospective studies with a larger sample size are needed to investigate the effect of preoperative HbA1c on intraoperative and postoperative complications in diabetic patients.

## Conclusions

The results of this study show that preoperative HbA1c above 7.5% is associated with an increased incidence of impaired intra- and postoperative glycaemic control; the incidence of hyperglycaemic episodes was higher than hypoglycemia. Postoperatively, the incidence of AKI, ICU admission, and duration of hospital stay were found to be higher in patients with preoperative HbA1c above or equal to 7.5%. However, the incidences were not different from those patients having preoperative HbA1c values <7.5%. Notably, the incidence of SSI was higher in patients with impaired perioperative glycemic control, suggesting the importance of perioperative glycemic control over the absolute preoperative HbA1c. Nevertheless, our finding is limited by the study's observational nature and the smaller sample. 
